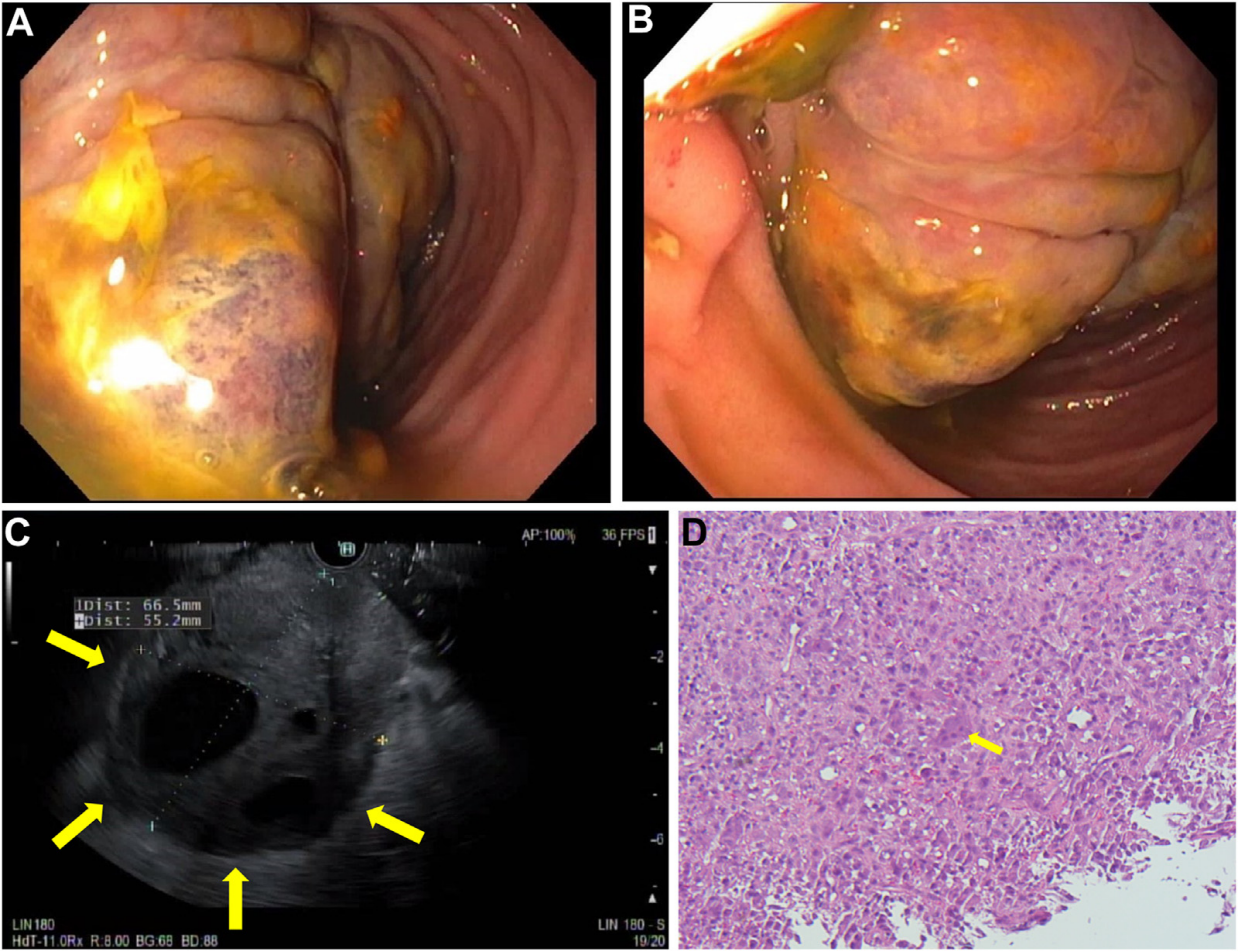# Undifferentiated Carcinoma of the Pancreas With Osteoclast-Like Giant Cells: A Grave Oncologic Diagnosis

**DOI:** 10.1016/j.gastha.2024.03.012

**Published:** 2024-03-22

**Authors:** Clive Jude Miranda, Eric John Dove, Farhan Azad

**Affiliations:** 1 Department of Gastroenterology, Hepatology, and Nutrition, University at Buffalo, Buffalo, New York; 2 Department of Pathology, University at Buffalo, Buffalo, New York; 3 Department of Hematology and Oncology, University at Buffalo, Buffalo, New York

A 51-year-old male with no gastrointestinal history presented with a 50lb weight loss, severe abdominal pain, and vomiting. Physical exam revealed a tender, firm epigastric nodule, and bloodwork was grossly normal, including CA-19-9 and carcinoembryonic antigen markers. Computed tomography revealed fat stranding around the pancreatic head and portal vein, superior mesenteric vein thrombosis, and peripancreatic adenopathy. There was a large mass in the third portion of the duodenum encasing branches of the superior mesenteric artery with gastric dilatation. An esophagogastroduodenoscopy with endoscopic ultrasound showed a 67 × 60 mm fungating villous mass with cystic components in D3 extending proximally to the stomach with intrinsic stenosis of the second portion of the duodenum (Figure A–C). Biopsy showed undifferentiated carcinoma of the pancreas with osteoclast-like giant cells (Figure D), which stained negative for CK7/CAM5.2 (seen in pancreatic ductal adenocarcinoma) and positive for CD68/vimentin (seen in osteoclast-like giant cells). Given his poor prognosis, a shared decision was made to transition him to hospice care. Undifferentiated carcinoma of the pancreas with osteoclast-like giant cells accounts for less than 1% of all pancreatic malignancies, is typically diagnosed at advanced stages, and is thereby frequently unresectable. Surgical resection is often first performed; however, optimal management is still consensus.

**Figure:**